# Positive association between the ratio of triglycerides to high-density lipoprotein cholesterol and diabetes incidence in Korean adults

**DOI:** 10.1186/s12933-021-01377-5

**Published:** 2021-09-09

**Authors:** Joungyoun Kim, Sang-Jun Shin, Ye-Seul Kim, Hee-Taik Kang

**Affiliations:** 1 College of Nursing, Mo-Im Kim Nursing Research Institute, Yonsei University, 50-1, Yonsei-Ro, Seodaemun-gu, Seoul, 03722 Republic of Korea; 2 Department of Information and Statistics, Chungbuk National University, 1 Chungdae-ro, Seowon-gu, Cheongju, Chungbuk 28644 Republic of Korea; 3 Department of Family Medicine, Chungbuk National University Hospital, 776 1-Soonwhan-ro, Seowon-gu, Cheongju, 28644 Republic of Korea; 4 Department of Family Medicine, Chungbuk National University College of Medicine, 1 Chungdae-ro, Seowon-gu, Cheongju, Chungbuk 28644 Republic of Korea

**Keywords:** Cholesterol, Triglycerides, Lipoproteins, HDL, Insulin resistance, Metabolic syndrome, Cardiometabolic risk factors, Diabetes mellitus

## Abstract

**Background:**

Insulin resistance is associated with the incidence of diabetes and cardiovascular diseases such as myocardial infarction. The ratio of triglycerides (TG) to high-density lipoprotein cholesterol (HDL-C) (TG/HDL-C ratio) is positively correlated with insulin resistance. This study aimed to investigate the relationship between the TG/HDL-C ratio and the incidence of diabetes in Korean adults.

**Methods:**

This retrospective study used data from the National Health Insurance Service-National Health Screening Cohort. The TG/HDL-C ratio was divided into three tertiles, the T_1_, T_2_, and T_3_ groups, based on sex. We estimated the hazard ratios (HRs) and 95% confidence intervals (CIs) for diabetes using multivariate Cox proportional hazards regression analyses.

**Results:**

A total of 80,693 subjects aged between 40 and 79 years were enrolled. The median follow-up period was 5.9 years. The estimated cumulative incidence of diabetes in the T_1_, T_2_, and T_3_ groups was 5.94%, 8.23%, and 13.50%, respectively, in men and 4.12%, 4.72%, and 6.85%, respectively, in women. Compared to T_1_, the fully adjusted HRs (95% CIs) of the T_2_ and T_3_ groups for new-onset diabetes were 1.17 (1.06–1.30) and 1.47 (1.34–1.62), respectively, in men and 1.20 (1.02–1.42) and 1.52 (1.30–1.78), respectively, in women.

**Conclusions:**

Increased TG/HDL-C ratio was significantly associated with a higher risk of new-onset diabetes in both sexes.

**Supplementary Information:**

The online version contains supplementary material available at 10.1186/s12933-021-01377-5.

## Introduction

Diabetes mellitus (DM) is a group of metabolic disorders characterized by a chronic hyperglycemic condition resulting from defects in glucose metabolism. This non-communicable disease is one of the largest global health concerns, imposing a heavy burden on public health and socio-economic development. Patients with type 2 diabetes are at a higher risk for cardio-cerebrovascular diseases, dementia, and some malignant neoplasms than those without diabetes [1]. According to the Global Burden of Disease Study 2017, the incidence and prevalence of diabetes increased significantly from 1990 to 2017. In 2017, 22.9 million new cases and 1.37 million diabetes-related deaths globally, and 476.0 million patients continued to suffer from this disease [2]. In the US, patients with diabetes pay higher medical costs than those without the disease [3]. In addition to the direct medical expenditure, diabetes increases indirect costs due to increased absenteeism, lower productivity at work, disease-related disability, and premature deaths [3]. The prevalence of diabetes in Korea has risen consistently over the past seven years, from 11.8% in 2012 to 13.8% in 2018, based on data from the 2012–2018 Korean National Health and Nutrition Examination Survey [4].

Various etiologies such as age, race, geography, socio-economic status, genetics, and health behaviors are involved in the development of diabetes [1]. In addition, impaired insulin secretion and insulin resistance may play an important role in developing the disease [5]. Thus, measurement of insulin resistance is essential to detect impaired glycemic control in the early phase of diabetes. The fasting insulin resistance index (FIRI) or homeostatic model assessment-insulin resistance (HOMA-IR) are widely used to measure insulin resistance [6]. However, using these indices is difficult in a clinical setting considering the complex calculation and low accuracy depending on the individual’s adiposity [7]. Several studies have suggested that the ratio of triglycerides (TG) to HDL cholesterol (HDL-C) (TG/HDL-C ratio), which is commonly used in clinical settings, is a more appropriate and simple indicator of insulin resistance and is associated with cardiovascular risk [8, 9]. If the TG/HDL-C ratio is well correlated with the incidence of diabetes, early detection or intervention based on this ratio can reduce the progression from normoglycemia or prediabetes to diabetes [10]. However, there is a lack of prospective evidence to investigate the association between the TG/HDL-C ratio and the development of diabetes, despite some cross-sectional studies in Korea [11].

Thus, this study aimed to investigate the incidence risk of diabetes based on the TG/HDL-C ratio in Korean adults without a history of diabetes, using data from the Korean National Health Insurance Service (NHIS)-Health Screening (HEALS) cohort.

## Methods

### Formation of cohort data and study population

We extracted the data for this study from the Korean NHIS-HEALS cohort database. The database included 514,794 individuals, who randomly sampled 10% of the total population of 5.1 million. The individuals in this cohort had undergone the national general health screening between 2002 and 2003, were aged between 40 and 79 years in 2002, and were followed up through 2015.

The cohort data contain information, including that death, healthcare usage, and health screening. The variables from the NHIS were income-based insurance contributions (a proxy for income), demographic variables, date of death, cause of death, prescription records, and disease diagnosis codes. The variables from the health screening database were the known risk factors for diabetes, based on self-questionnaires (cigarette smoking status, alcohol drinking status, physical activity level, personal medical history, and family medical history) and bio-clinical laboratory results. This cohort was followed up from 2002 to 2015; however, lipid profile parameters such as serum TG, HDL-C, and low-density lipoprotein cholesterol (LDL-C) levels were measured from 2009; therefore, we set 2009 and 2010 as the baseline years.

**Fig. 1:**
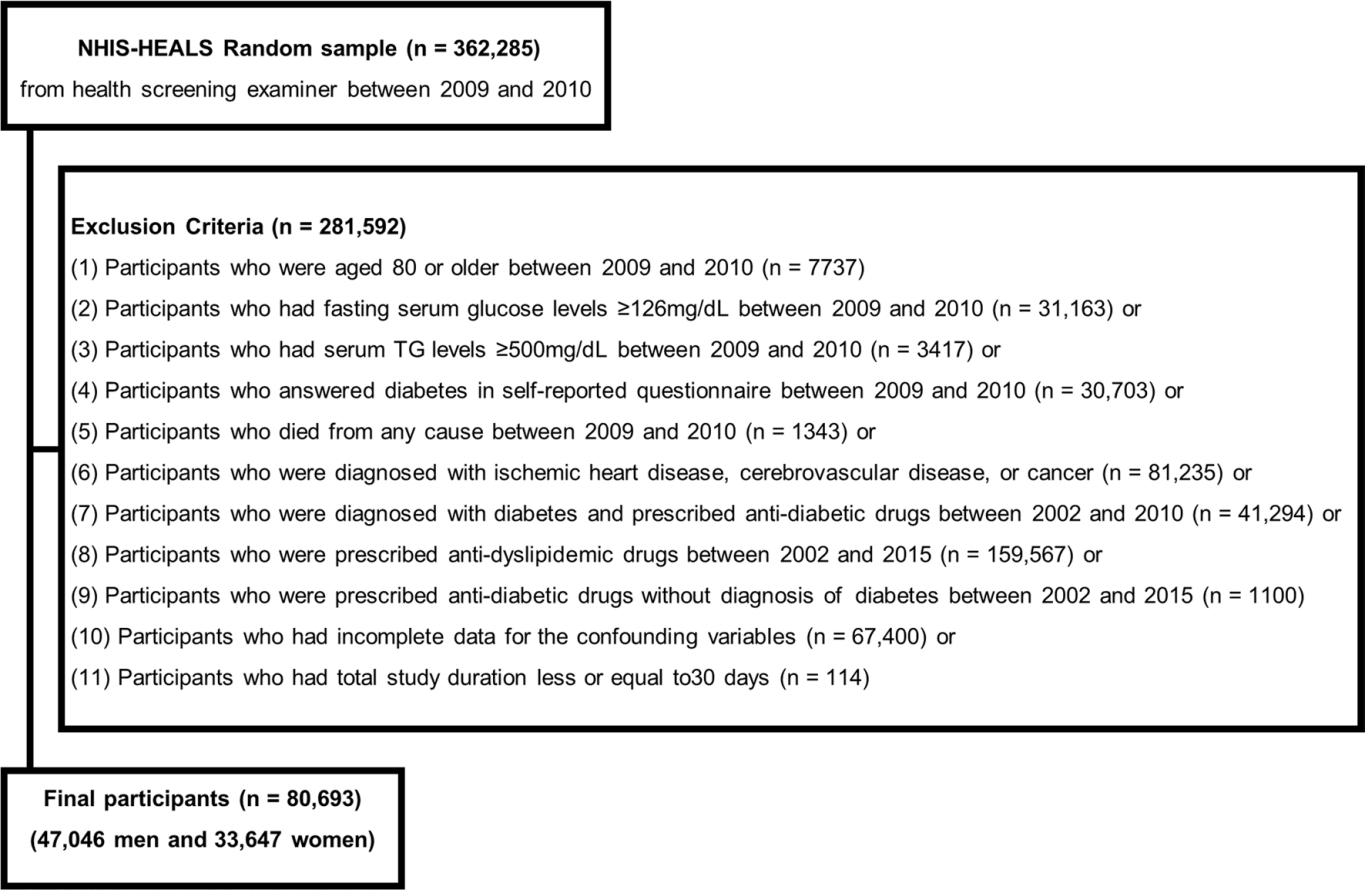
Flowchart of the inclusion and exclusion criteria

Figure 1 presents the flowchart of the inclusion and exclusion criteria for this study. Of the initial 362,285 participants at baseline, 281,592 were excluded based on the following exclusion criteria: (1) participants who were aged 80 or older between 2009 and 2010 (n  =  7737); (2) those who had fasting serum glucose levels  ≥  126 mg/dL between 2009 and 2010 (n  =  31,163); (3) those who had serum TG levels  ≥  500 mg/dL between 2009 and 2010 (n  =  3417); (4) those who answered diabetes in self-reported questionnaire between 2009 and 2010 (n  =  30,703); (5) those who died from any cause between 2009 and 2010 (n  =  1343); (6) those who were diagnosed with ischemic heart diseases (I20–I25), cerebrovascular diseases (I60–I69), or cancers (C00–C97) based on the 10th edition of the International Classification of Diseases (ICD-10) between 2002 and 2010 (n  =  81,235); (7) those who were diagnosed with diabetes (E10–E14) and prescribed any anti-diabetic drugs between 2002 and 2010 (n  =  41,294); (8) those who were prescribed anti-dyslipidemic drugs those who were prescribed anti-dyslipidemic drugs (statins, ezetimibe, fibric acids, cholesterol sequestrants, and omega-3 fatty acids) between 2002 and 2015 (n  =  159,567); (9) those who were prescribed anti-diabetic drugs without diagnosis of diabetes between 2002 and 2015 (n  =  1100); (10) those who had incomplete data for the confounding variables (n  =  67,400); or (11) those who had total study duration less or equal to 30 days (n  =  114). Participants were sequentially excluded based on the criteria mentioned above, and they were not mutually exclusive. After full exclusion, we included 80,693 participants (47,046 men and 33,647 women) in the final analyses.

The Institutional Review Board of Chungbuk National University approved the present study (CBNU-202011-HR-0179), which adhered to the principles of the Declaration of Helsinki (1975).

### The definitions of diabetes, subject groups, and study date

We defined diabetes as the presence of one of the following conditions: (1) a record of diabetes diagnosis (ICD-10 code E11–14) and prescription of any anti-diabetic drug (insulin, sulfonylurea, metformin, thiazolidinedione, dipeptidyl peptidase-4 inhibitor, α-glucosidase inhibitor, sodium-glucose cotransporter-2 inhibitor, glucagon-like peptide (GLP)-1 agonist, and/or others), or (2) a fasting blood glucose level  ≥  126 mg/dL on health screening. The date of incidence of diabetes was defined as the earliest between the two dates that satisfied the above conditions.

Based on analysis of the risk of diabetes according to the TG/HDL-C ratio, the participants were divided into three groups: T_1_,  <  1.681 in men and  <  1.278 in women; T_2_, 1.681– <  2.951 in men and 1.278– <  2.142 in women; and T_3_,  ≥  2.951 in men and  ≥  2.142 in women.

The research start date was defined as the day of the first health examination between 2009 and 2010. For the participants diagnosed with diabetes between 2011 and 2015, the research end date was the initial diagnosis date of the disease. In cases where the participant died before a diagnosis of diabetes, the end date was defined as the date of death. Similarly, in cases where the participants had not died or had not been diagnosed with diabetes during the study period, the end date was the latest date of the last outpatient clinic visit, last health screening, or last when the prescribed medication was taken.

### Definition of covariates

In this study, analyses were conducted by controlling for confounding variables such as age, systolic blood pressure (SBP), body mass index (BMI) (kg/m^2^), serum fasting glucose, total cholesterol, and alanine aminotransferase (ALT) levels, personal history of hypertension, family history of diabetes, smoking status, alcohol consumption, physical activity, and household income levels between 2009 and 2010.

BMI was defined as body mass in kilograms divided by the square of height in meters. We collected data regarding hypertension, family history of diabetes, smoking status, alcohol consumption, and physical activity from self-reported questionnaires. Smoking status was categorized as non-smoker or ever-smoker. Non-smokers were defined as individuals who answered “No” to “Have you ever smoked 5 packs (100 cigarettes) or more in your lifetime?” in the self-reported questionnaires. Ever-smokers were defined as individuals who answered “Yes” to this question [12, 13]. Alcohol consumption was classified as rare (less than once a week), sometimes (once to twice per week), and often (three or more times per week) [14]. Physical activity was divided into three categories: rare (less than once a week of any intensity exercise), sometimes (not meeting the definition of the rare or regular categories), and regular (five or more times of walking or moderate-intensity exercise per week; three or more times of vigorous-intensity exercise per week; four or more times of walking [or moderate-intensity exercise] and vigorous-intensity exercise per week) [15]. We categorized monthly household income into three groups: low, 0–3rd deciles; middle, 4th–7th deciles; and high, 8th–10th deciles.

### Statistical analysis

Continuous variables are expressed as mean  ±  standard deviation (SD). Categorical variables are expressed as the number of subjects (percentage). For group comparison, analysis of variance (ANOVA) and Chi-square tests were used for the continuous and categorical variables, respectively.

To determine the association between the TG/HDL-C ratio and the incidence of diabetes, we estimated and compared diabetes-free survival rates using the Kaplan–Meier method based on the log-rank test. Cox proportional hazards regression models were adopted to calculate the hazard ratios (HRs) and 95% confidence intervals (95% CIs) after controlling for confounding factors. Four Cox proportional hazards regression models were considered: (1) Model 1: age only; (2) Model 2: age, smoking status, alcohol consumption, and physical activity; (3) Model 3: BMI, SBP, total cholesterol level, ALT level, history of hypertension, family history of diabetes, and household income in addition to the variables in Model 2; and (4) Model 4: fasting glucose level in addition to the variables in Model 3.

All p-values reported are two-sided, and statistical significance was set at p  <  0.05. The statistical packages SAS enterprise guide version 7.1 (SAS Inc., Cary, NC) and R studio version 3.3.3 were used to perform the study analyses.

## Results

The total study population was 80,693 (47,046 men and 33,647 women), and the median follow-up duration was 5.9 years. Table 1 shows the baseline characteristics of the study population based on the TG/HDL-C ratio. BMI, SBP, serum fasting glucose, total cholesterol, ALT levels, and percentage of hypertension increased the higher TG/HDL-C ratio in both sexes (Table 1). The higher tertile groups of the TG/HDL-C ratio had fewer regular alcohol drinkers and a less physically active population than in the lower tertile group (all p value  <  0.001). Men in the higher tertile groups were younger, while women were older. Household income, percentage of a family history of diabetes, and the number of ever-smokers were higher in the higher tertile groups than in the lower tertile group among men, while the variables were not statistically significant in women.

**Table 1:** Baseline characteristics of the study participants according to the TG/HDL-C ratio groups

Men	T_1_ (< 1.681)	T_2_ (1.681– < 2.951)	T_3_ (≥ 2.951)	p value
Number of subjects	15,692	15,675	15,679	NA
TG/HDL-C	1.17 ± 0.32	2.25 ± 0.36	4.84 ± 1.99	< 0.001
Age, yr	56.5 ± 7.8	55.8 ± 7.5	55.0 ± 7.1	< 0.001
BMI, kg/m^2^	22.7 ± 2.6	23.8 ± 2.6	24.6 ± 2.5	< 0.001
SBP, mmHg	122.9 ± 14.5	124.6 ± 14.3	126.0 ± 14.2	< 0.001
Fasting glucose, mg/dL	94.2 ± 11.1	95.4 ± 11.2	96.4 ± 11.5	< 0.001
Total cholesterol, mg/dL	186.6 ± 29.4	191.4 ± 30.7	195.3 ± 31.8	< 0.001
TG, mg/dL	71.3 ± 22.4	115.2 ± 24.9	205.6 ± 71.2	< 0.001
HDL-C, mg/dL	64.4 ± 38.8	51.4 ± 9.3	43.8 ± 8.6	< 0.001
ALT, IU/L	23.4 ± 17.4	25.4 ± 16.5	29.2 ± 19.9	< 0.001
Hypertension, N (%)	2086 (13.2)	2534 (16.2)	2618 (16.7)	< 0.001
Family history of diabetes, N (%)	832 (5.3)	906 (5.8)	1036 (6.6)	< 0.001
Ever-smokers, N (%)	9308 (59.3)	9981 (63.7)	10,728 (68.4)	< 0.001
Alcohol consumption, N (%)				< 0.001
Rare	5450 (34.7)	5584 (35.6)	5318 (33.9)	
Sometimes	6500 (41.4)	6553 (41.8)	6799 (43.4)	
Regular	3742 (23.8)	3538 (22.6)	3562 (22.7)	
Physical activity, N (%)				< 0.001
Rare	3288 (21.0)	3186 (20.3)	3302 (21.1)	
Sometimes	6903 (44.0)	7513 (47.9)	7751 (49.4)	
Regular	5501 (35.1)	4976 (31.7)	4626 (29.5)	
Household income, N (%)				< 0.001
Low	2301 (14.7)	2224 (14.2)	2064 (13.2)	
Middle	4790 (30.5)	4609 (29.4)	4585 (29.2)	
High	8601 (54.8)	8842 (56.4)	9030 (57.6)	

Women	T_1_ (< 1.278)	T_2_ (1.278– < 2.142)	T_3_ (≥ 2.142)	p value
Number of subjects	11,227	11,207	11,213	NA
TG/HDL-C	0.92 ± 0.24	1.67 ± 0.25	3.54 ± 1.58	< 0.001
Age, yr	54.5 ± 6.8	56.3 ± 7.7	58.3 ± 8.4	< 0.001
BMI, kg/m^2^	22.7 ± 2.7	23.4 ± 2.9	24.1 ± 2.9	< 0.001
SBP, mmHg	118.2 ± 14.6	120.6 ± 15.1	123.6 ± 15.6	< 0.001
Fasting glucose, mg/dL	91.1 ± 10.0	92.2 ± 10.1	93.2 ± 10.5	< 0.001
Total cholesterol, mg/dL	193.2 ± 29.9	194.9 ± 30.2	198.8 ± 31.3	< 0.001
TG, mg/dL	60.9 ± 19.8	94.3 ± 19.1	163.5 ± 58.4	< 0.001
HDL-C, mg/dL	70.8 ± 47.7	56.8 ± 9.7	47.9 ± 9.1	< 0.001
ALT, IU/L	19.4 ± 19.7	20.2 ± 14.8	21.9 ± 14.8	< 0.001
Hypertension, N (%)	1288 (11.5)	1769 (15.8)	2299 (20.5)	< 0.001
Family history of diabetes, N (%)	912 (8.1)	861 (7.7)	815 (7.3)	0.056
Ever-smokers, N (%)	194 (1.7)	207 (1.8)	238 (2.1)	0.085
Alcohol consumption, N (%)				< 0.001
Rare	8979 (80.0)	9484 (84.6)	9736 (86.8)	
Sometimes	1905 (17.0)	1429 (12.8)	1255 (11.2)	
Regular	343 (3.1)	294 (2.6)	222 (2.0)	
Physical activity, N (%)				< 0.001
Rare	3020 (26.9)	3382 (30.2)	3602 (32.1)	
Sometimes	4914 (43.8)	4736 (42.3)	4723 (42.1)	
Regular	3293 (29.3)	3089 (27.6)	2888 (25.8)	
Household income, N (%)				0.080
Low	2906 (25.9)	2839 (25.3)	2797 (24.9)	
Middle	3925 (35.0)	4021 (35.9)	4125 (36.8)	
High	4396 (39.2)	4347 (38.8)	4291 (38.3)	

Values are presented as n (%) or mean  ±  standard deviation

*SBP* systolic blood pressure; *BMI* body mass index; *TG* triglyceride; *HDL-C* HDL cholesterol; *TG/HDL-C ratio* the ratio of TG to HDL-C; *ALT* alanine aminotransferase

**Fig. 2:**
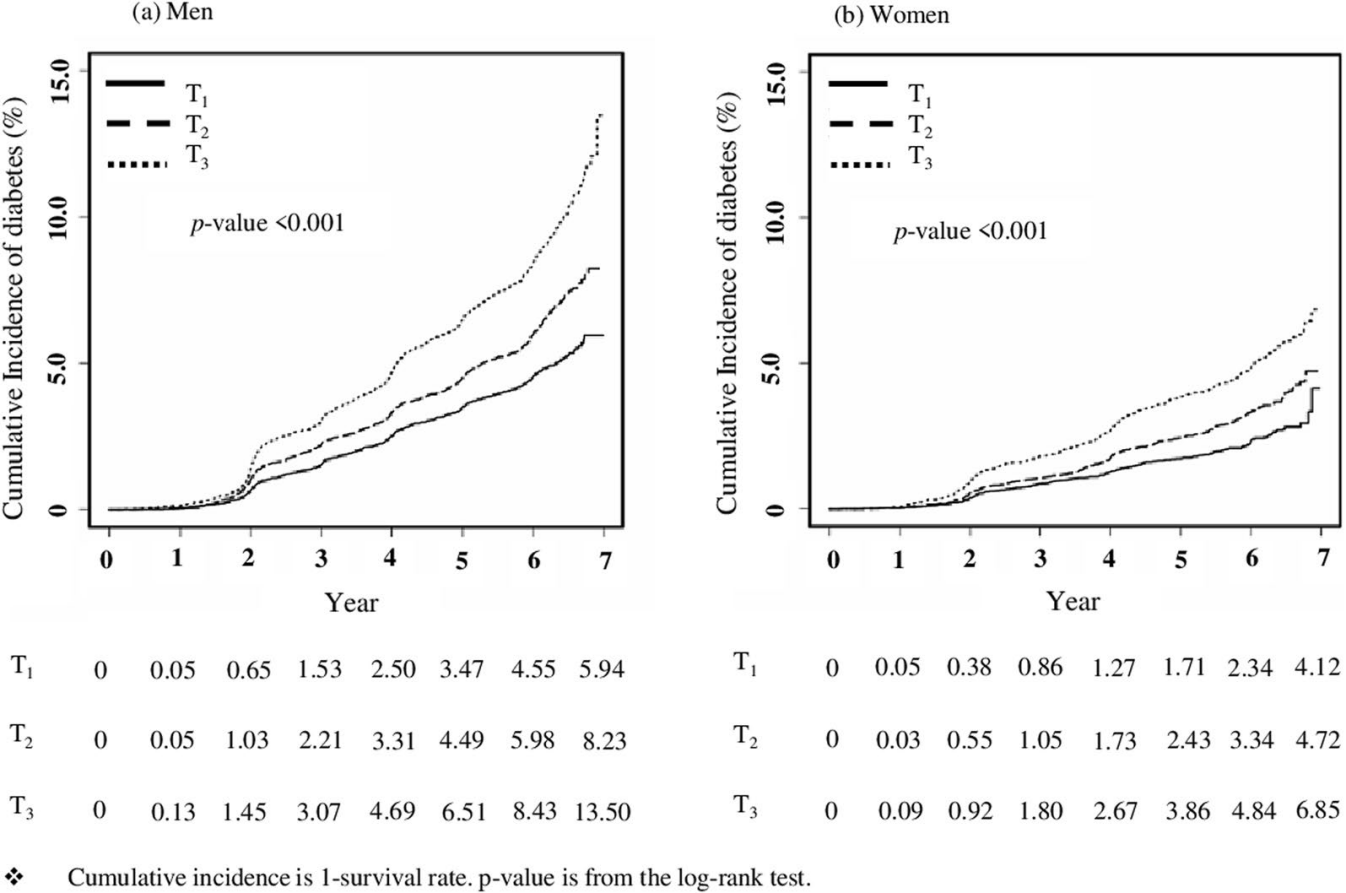
The estimated cumulative incidence of type 2 diabetes mellitus

Figure 2 shows the estimated cumulative incidence rates of diabetes based on the Kaplan–Meier survival curve. Cumulative incidence was the highest in T_3_ and lowest in T_1_ in both sexes (log-rank test p values  <  0.001). A total of 4093 cases of diabetes were observed, accounting for 5.1% of the total participants. At the end of the follow-up period, the estimated cumulative incidences of diabetes from T_1_ to T_3_ were 5.94%, 8.23%, and 13.50%, respectively, in men and 4.12%, 4.72%, and 6.85%, respectively, in women.

Table 2 shows the results of the Cox proportional hazards regression models (Table 2). We used Cox proportional hazards regression models to quantify the risk of diabetes according to the TG/HDL-C ratio. Compared with T_1_, HRs (95% CIs) for the incidence of diabetes in T_2_ and T_3_ were 1.35 (1.23–1.49) and 1.93 (1.76–2.12), respectively, in men and 1.39 (1.19–1.64) and 2.02 (1.73–2.35), respectively, in women after adjusting for age only (Model 1). After adjusting for the variables included in Model 3, HRs (95% CIs) for diabetes incidence of T_2_ and T_3_ were 1.22 (1.10–1.34) and 1.57 (1.42–1.73), respectively, in men and 1.27 (1.08–1.49) and 1.64 (1.41–1.92), respectively, in women (Model 3). Furthermore, we adjusted fasting glucose levels to minimize the effects of baseline glucose levels in Model 4. After full adjustment, the HRs (95% CIs) for the incidence of diabetes in T_2_ and T_3_ were 1.17 (1.06–1.30) and 1.47 (1.34–1.62), respectively, in men and 1.20 (1.02–1.42) and 1.52 (1.30–1.78), respectively, in women (Model 4).

**Table 2:** Cox proportional hazards regression results for diabetes incidence according to the TG/HDL-C ratio groups

HRs (95% CIs)	Men			Women		
	T_1_ (< 1.681)	T_2_ (1.681– < 2.951)	T_3_ (≥ 2.951)	T_1_ (< 1.278)	T_2_ (1.278– < 2.142)	T_3_ (≥ 2.142)
Model 1	1	1.35 (1.23–1.49)	1.93 (1.76–2.12)	1	1.39 (1.19–1.64)	2.02 (1.73–2.35)
Model 2	1	1.36 (1.23–1.50)	1.93 (1.78–2.12)	1	1.40 (1.19–1.65)	2.04 (1.75–2.37)
Model 3	1	1.22 (1.10–1.34)	1.57 (1.42–1.73)	1	1.27 (1.08–1.49)	1.64 (1.41–1.92)
Model 4	1	1.17 (1.06–1.30)	1.47 (1.34–1.62)	1	1.20 (1.02–1.42)	1.52 (1.30–1.78)

Model 1: adjusted for age

Model 2: adjusted for smoking status, alcohol consumption, and physical activity in addition to Model 1

Model 3: adjusted for body mass index, systolic blood pressure, serum total cholesterol, alanine aminotransferase, past hypertension history, family history of diabetes, and monthly household income, in addition to Model 2

Model 4: adjusted for fasting glucose levels, in addition to Model 3

We examined the area under the curves (AUCs) of fasting glucose, TG/HDL ratio, and the combination of TG/HDL-C ratio and fasting glucose (Additional file 1: Figure S1). In all models, fasting glucose had a better predictive power than the TG/HDL-C ratio. The AUCs of the receiver operating curve (ROC) analyses were 0.708, 0.604, 0.718, respectively, in all participants; 0.686, 0.586, and 0.695, respectively, in men; and 0.732, 0.596, and 0.738, respectively, in women. Delong’s test was performed for all possible pairwise comparisons to test for significant differences between the ROC curves. In each comparison, the p value was less than 0.001.

## Discussion

This retrospective study demonstrated that an elevated TG/HDL-C ratio was positively associated with the incidence of diabetes in the Korean population, based on the Korean NHIS-HEALS data. Our study provides additional information that high TG and low HDL-C levels, as surrogate markers of insulin resistance, may predict the risk of diabetes in primary clinical settings.

Insulin resistance is a crucial pathophysiological parameter of metabolic syndrome, which is a cluster of metabolic risk factors associated with an increased risk for type 2 diabetes and cardiovascular diseases [16]. Insulin resistance means that insulin stimuli fail to induce an appropriate biologic response, such as glycemic control. Consequently, the human body increases the production of pancreatic insulin and results in hyperinsulinemia to compensate for the dysfunction. The exact measurement of insulin resistance preceding type 2 diabetes is very important to prevent its harmful consequences, including diabetes and cardiovascular diseases [17, 18]. Several methods have been suggested to measure insulin resistance. The hyperinsulinemic-euglycemic glucose clamp technique is considered the gold standard method to assess insulin resistance. However, it is not easy to apply this technique in real-world clinical settings considering the complex measurement procedure [19]. HOMA-IR is commonly used to assess insulin resistance [20]; however, it has limited applicability. Its calculation involves a complex equation and measured fasting insulin levels. The TG/HDL-C ratio is another reliable surrogate marker to assess insulin resistance [21, 22]. Lipid profile parameters such as TG and HDL-C are inexpensive laboratory tests; therefore, they are more commonly used to assess cardiovascular risk than insulin. In addition, the TG/HDL-C ratio can be calculated easily by dividing TG with HDL-C. A cross-sectional study reported the correlation between the TG/HDL-C ratio and insulin resistance regardless of waist circumference in the Korean adult population [11]. Although our results are consistent with several previous studies in that the TG/HDL-C ratio was significantly associated with cardiovascular risk factors and the incidence of diabetes [23–25], there remain contradictory claims that the ratio is not a reliable indicator of insulin resistance. Thus, further research is needed [26].

The mechanism by which a high TG/HDL-C ratio increases the incidence of diabetes is unclear, although the ratio appears to be a relatively reliable indicator of insulin resistance. Previous studies have suggested dyslipidemia as a causal factor of insulin resistance [27]. An increase in TG and decreased HDL-C levels through genetic variants in lipid-related genes could cause insulin resistance [28]. It results in compensatory hyperinsulinemia, leading to aggravation of hypertriglyceridemia. Other studies have reported that lipotoxicity might play a role in insulin resistance and pancreatic β-cell dysfunction by producing inflammatory cytokines, mitochondrial oxidation, and the protein kinase C and c-Jun NH2-terminal kinase (JNK)-1 pathways in non-adipose tissue organs [29–31]. In addition, it has been suggested that hypertriglyceridemia and insulin resistance are linked through the cholesteryl ester transfer protein (CETP). The CETP is a glycoprotein that regulates the equilibrium of lipoprotein fractions by promoting the bidirectional transfer of cholesteryl esters (CE) and TG [32]. In previous studies on hypertriglyceridemia patients, CETP activity was elevated. This elevated activity caused the conversion of TG to TG-enriched LDL and HDL to small-dense HDL, resulting in decreased HDL-C levels [33, 34]. Although the mechanism of the association between CETP and insulin resistance has not yet been precisely elucidated, previous studies showed that when a CETP inhibitor was used, HDL-C increased, and the HOMA-IR and glycemic control in type 2 diabetic patients were improved [35, 36]. This study explained that the CETP inhibitor possibly increased the insulin-stimulated glucose uptake in the skeletal muscles, liver, and heart, improving glucose homeostasis [35]. These results implied the possibility that CETP may also affect glucose metabolism and insulin action regulation in addition to its action in lipoprotein metabolism.

Insulin resistance tends to increase with age, obesity, and an unhealthy lifestyle, such as limited physical activity [37–39]. This trend was expected to be observed in the TG/HDL-C ratio as well, which indirectly reflects insulin resistance. Contrary to expectations, in our study, the men in the group with a higher TG/HDL-C ratio were younger and had higher household incomes. The group with a lower TG/HDL-C ratio had a higher percentage of participants (both men and women) who consumed alcohol regularly. Additional adjustment for fasting blood glucose levels at baseline was performed. Fasting blood glucose levels are one of the most critical risk factors for predicting the development of diabetes. Even after fully adjustment, the TG/HDL-C ratio was significantly associated with the incidence of diabetes in both men and women. This result supports the findings reported by Qi et al. [40] in that the TG/HDL-C ratio can increase the risk of type 2 diabetes independent of insulin resistance, similar to genetic predisposition.

Our study has several limitations. First, because the NHIS-HEALS cohort did not collect serum insulin levels, we could not validate the correlation between the TG/HDL-C ratio and insulin resistance. By validating this correlation, it can be concluded that the positive association between the TG/HDL-C ratio and diabetes risk results from insulin resistance. However, increased TG/HDL-C ratio may influence the occurrence of diabetes through its unique properties such as genetic predisposition, apart from insulin resistance. Second, the type of diabetes, the diagnosis time, and the incidence may differ from the actual rate due to the limitations of cohort data. Although this study tried to confirm type 2 DM, the incidence of diabetes was defined based on the fasting glucose level during the biannual health screening tests or the prescription of anti-diabetic medications combined with clinical diagnostic codes of diabetes-related diseases. Diabetes was diagnosed based on the following criteria: (1) fasting blood glucose  ≥  126 mg/dL after at least 8-h fasting; (2) glycated hemoglobin (HbA1C  ≥  6.5% (48 mmol/mol); 2-h blood glucose  ≥  200 mg/dL during an oral glucose tolerance test; and random blood glucose  ≥  200 mg/dL with classic hyperglycemic symptoms [41]. This operational definition of diabetes adopted in this study might have led to inaccurate results. In addition, the actual diagnosis or onset of diabetes could be different from the real-world data. However, the incidence rate of type 1 DM in Korean adults over 20 years of age is 2.99 per 1,00,000 [42]. As such, the possibility of type 1 DM occurrence in this study is considered low.

Third, we could not include other risk factors for diabetes due to limited data availability. In particular, dietary patterns in which foods with a high glycemic index or load are consumed can increase the risk of diabetes [43, 44]. Unfortunately, the NHIS-HEALS cohort did not include this information. Fourth, there is no consensus regarding the normal range or cut-off value of the TG/HDL-C ratio. Previous studies have shown that the TG/HDL-C ratio value differed according to age, sex, diet, country, race, and culture [45, 46]. Wakabayashi et al.’s studies reported TG/HDL-C ratio varied age and sex and showed a J-shaped relationship with alcohol intake in a hypertensive population [47, 48]. Another study reported that diet significantly altered the lipid profile [49]. Also, since this study demonstrated a positive correlation in a dose-dependent manner, we believe that the TG/HDL-C ratio is a risk factor for diabetes. In addition, the TG/HDL-C ratio can be easily measured in primary clinical settings and can provide additional information regarding cardiovascular diseases.

Despite several limitations, this study has certain advantages over other studies. First, the Korean NHIS-HEALS cohort represents the entire Korean population. A retrospective cohort study was recently published to present the association between the TG/HDL-C ratio and the risk of diabetes [50]. However, the study as mentioned earlier, might not represent the characteristics of the general Korean population because it analyzed a cohort consisting of residents in two regions. In contrast, since our study included a sample of NHIS-insured individuals, the results can be generalized to a significantly larger population. Second, we adopted a relatively conservative approach to minimize unexpected biases. Individuals who had taken any lipid-lowering drugs, such as statins, ezetimibe, fibric acid, cholesterol sequestrants, and omega-3 fatty acids, were excluded by using the ATC code from this study. For instance, most statins that reduce cholesterol levels and prevent cardiovascular diseases can increase blood glucose levels and cause new-onset diabetes [51]. Previous studies have reported that statin treatment affects the calcium channel of the pancreatic β-cells by altering insulin secretion or decreasing glucose transporter 4 translocation, leading to hyperglycemia and hyperinsulinemia, which can be related to the development of diabetes [52]. And, omega-3 fatty acids could affect the study results because they lower the serum TG level by increasing β-oxidation and, decreasing the hepatic fatty acids synthesis [53, 54]. Third, the median follow-up duration of 5.9 years is relatively long. Chronic diseases, such as diabetes, require a long time from exposure to clinical onset. Therefore, a sufficient follow-up period is essential to analyze the association in a favorable manner.

The normal range or cut-off value of the TG/HDL-C ratio for increased risk of diabetes in healthy adults or diabetic risk groups has not been standardized. Through the ROC analysis results in this study, the predictive power of diabetes using the TG/HDL-C ratio alone was lower than that of the fasting glucose. However, compared to the model using fasting glucose alone, the model’s predictive power improved after combining the TG/HDL-C ratio and fasting glucose. So, the TG/HDL-C ratio, a relatively simple test to perform in the clinic, can be used as an additional diagnostic tool. Assessment of this ratio might help identify the risk group for diabetes transition and plan lifestyle modifications.

In conclusion, elevated TG/HDL-C ratio was positively associated with increased diabetes incidence in the Korean population.

## Supplementary Information

**Additional file 1: Figure S1.** ROC curve analysis of TG/HDL-C ratio and fasting glucose as a predictor of DM incidence.

## Data Availability

Not applicable.

## Abbreviations

ALT: Alanine aminotransferase
ANOVA: Analysis of variance
AUC: Area under the curves
BMI: Body mass index
CE: Cholesteryl esters
CETP: Cholesteryl ester transfer protein
CI: Confidence interval
DM: Diabetes mellitus
FIRI: Fasting insulin resistance index
GLP: Glucagon-like peptide
HDL-C: High-density lipoprotein cholesterol
HR: Hazard ratio
HOMA-IR: Homeostatic model assessment-insulin resistance
HEALS: Health screening
ICD: International classification of diseases
LDL-C: Low-density lipoprotein cholesterol
NHIS: National Health Insurance Service
ROC: Receive operator curve
SBP: Systolic blood pressure
SD: Standard deviation
SNP: Single nucleotide polymorphism
TG: Triglycerides
TG/HDL-C ratio: The ratio of triglyceride to high-density lipoprotein cholesterol
