# Supplementary figures and images for: Positive association between the ratio of triglycerides to high-density lipoprotein cholesterol and diabetes incidence in Korean adults
Source: Cardiovasc Diabetol. 2021 Sep 9;20:183. doi: 10.1186/s12933-021-01377-5 (PMC8431895; doi:10.1186/s12933-021-01377-5)

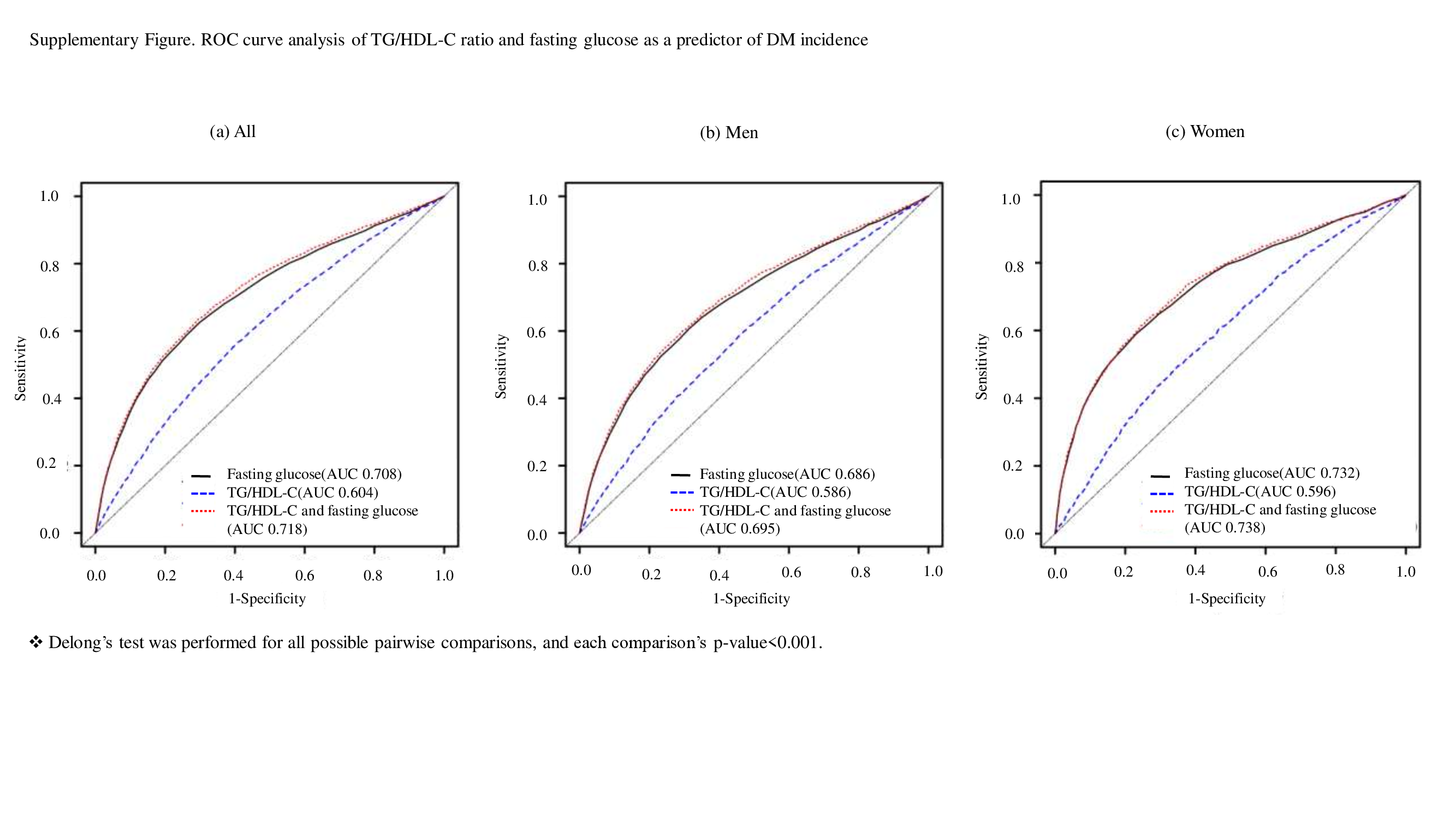

Supplement: Supplementary file 1 — Additional file 1: Figure S1. ROC curve analysis of TG/HDL-C ratio and fasting glucose as a predictor of DM incidence. [file 12933_2021_1377_MOESM1_ESM.tiff]
